# Seasonal assessment of selected trace elements in grass carp (*Ctenopharyngodon idella*) blood and their effects on the biochemistry and oxidative stress markers

**DOI:** 10.1007/s10661-023-12152-2

**Published:** 2023-11-23

**Authors:** Anton Kovacik, Eva Tvrda, Marian Tomka, Norbert Revesz, Julius Arvay, Martin Fik, Lubos Harangozo, Lukas Hleba, Eva Kovacikova, Tomas Jambor, Miroslava Hlebova, Jaroslav Andreji, Peter Massanyi

**Affiliations:** 1 Institute of Applied Biology, Faculty of Biotechnology and Food Sciences, Slovak University of Agriculture in Nitra, Tr. A. Hlinku 2, 949 76 Nitra, Slovakia; 2 Institute of Biotechnology, Faculty of Biotechnology and Food Sciences, Slovak University of Agriculture in Nitra, Tr. A. Hlinku 2, 949 76 Nitra, Slovakia; 3 DSM Nutritional Products Inc. Hungary Kft, Japán Fasor 4, 2367 Újhartyán, Hungary; 4 Institute of Food Sciences, Faculty of Biotechnology and Food Sciences, Slovak University of Agriculture in Nitra, Tr. A. Hlinku 2, 949 76 Nitra, Slovakia; 5 Institute of Animal Husbandry, Faculty of Agrobiology and Food Resources, Slovak University of Agriculture in Nitra, Tr. A. Hlinku 2, 949 76 Nitra, Slovakia; 6 AgroBioTech Research Centre, Slovak University of Agriculture in Nitra, Tr. A. Hlinku 2, 949 76 Nitra, Slovakia; 7 Department of Biology, Institute of Biology and Biotechnology, Faculty of Natural Sciences, University of Ss. Cyril and Methodius, Nám. J. Herdu 2, 917 01 Trnava, Slovakia

**Keywords:** Biomarkers, Reactive Oxygen Species, Trace elements, Metals, Biomonitoring, Freshwater fish

## Abstract

Environmental pollution by anthropogenic activity is still a highly relevant global problem. Aquatic animals are a specifically endangered group of organisms due to their continuous direct contact with the contaminated environment. Concentrations of selected trace elements in the grass carp (*Ctenopharyngodon idella*) (*n* = 36) blood serum/clot were monitored. Possible effects of the elements on selected biochemical and oxidative markers were evaluated. The concentrations of trace elements (Al, Ba, Be, Bi, Cd, Co, Cr, Cu, Fe, Ga, Mn, Mo, Ni, Pb, Sr, Tl, and Zn) were analysed in the fish blood serum and blood clot by inductively coupled plasma optical emission spectrometry (ICP OES). A general scheme of decreasing concentrations of trace elements in the blood serum samples was: Zn ˃ Fe ˃ Sr ˃ Ba ˃ Ni ˃ Al ˃ Cu ˃ Be ˃ Co; < LOQ (below limit of quantification): Bi, Cd, Cr, Ga, Mn, Mo, Pb, Tl; and in the case of the blood clot, the scheme was as follows: Fe ˃ Zn ˃ Sr ˃ Al ˃ Ni ˃ Ba ˃ Cu ˃ Be ˃ Co ˃ Mn; < LOQ (below limit of quantification): Bi, Cd, Cr, Ga, Mo, Pb, Tl. Significant differences among the seasons were detected. The Spearman *R* correlation coefficients and linear or non-linear regression were used to evaluate direct relationships between trace elements and selected blood biomarkers. The correlation analysis between biochemical parameters (Na, K, P, Mg, AST, ALT, ALP, GGT, TAG, TP, urea, glucose) and trace elements (Al, Ba, Be, Cu, Fe, Ni, Sr, and Zn) concentrations confirmed statistically significant interactions in both seasons (summer and autumn). The regression analysis between oxidative stress markers (ROS, GPx, creatinine, uric acid, and bilirubin) and elements (Al, Ba, Co, Cu, Fe, Ni, and Sr) content confirmed statistically significant interactions. The results point to numerous connections between the observed elements and the physiological parameters of freshwater fish.

## Introduction

Environmental pollution by anthropogenic activity is a pressing global problem. Aquatic animals are a specifically endangered group of organisms due to their continuous direct contact with the contaminated environment. The water ecosystem is in fact absolutely connected with the waste systems of industry, the energy sector, agriculture, or households. Monitoring environmental pollutants in relation to aquatic organisms’ health status is an important activity that could reflect the effects and risks stemming from contaminants on animal organisms. Trace elements, pesticides, and microplastics are among the most widespread environmental contaminants today (Clasen et al., 2018; Lushchak, 2011; Shahjahan et al., 2022a, 2022b).

Assessment of trace elements in the blood and tissues of fish can prevent any potential food chain risks while serving as an early detection of contamination (Ansel & Benamar, 2018; Hansson et al., 2020; Hinojosa-Garro et al., 2020; Lakra et al., 2022; Monferrán et al., 2016; Ruas et al., 2008). On the other hand, increased concentrations of toxic as well as essential elements result in various physiological and health alterations (Emenike et al., 2022; Khan et al., 2020; Öner et al., 2008; Shahjahan et al., 2022a, 2022b), defects particularly to the liver, and kidney (Banday et al., 2019; Erdoğan et al., 2021; Lakra et al., 2022; Tlenshieva et al., 2022), and the reproductive system (Gárriz et al., 2019; Kovacik et al., 2018; Kročková et al., 2013; Özgür et al., 2019) in animal species. Blood parameters, such as serum chemistry and enzymatic markers, may provide us with the physiological response of the organism in connection with metals (Gopal et al., 1997; Phoonaploy et al., 2019). Another important system for the assessment of environmental contamination or intoxication by trace elements are oxidative stress markers (El-Sharawy et al., 2022; Giarratano et al., 2016; Kovacik et al., 2019; Lushchak, 2011). The production of reactive oxygen species (ROS) or ROS-induced modification of lipids and proteins leads to lipid peroxidation and the production of carbonyl proteins, which allows us to evaluate the range of pathological processes related to free radicals (Lushchak, 2011, 2014). A subsequent evaluation of the total antioxidant capacity (TAC), enzymatic and non-enzymatic endogenous oxidative status markers may indicate an overall association of environmental pollutants and trace elements on the animal organism (Lushchak, 2016; Shahjahan et al., 2022a, 2022b; Tvrdá et al., 2016a, 2016b).

Based on the above, we focused on several objectives in the present study. In our conditions, we chose to collect samples in two seasons: summer and autumn. The primary goal was to assess the levels of selected trace elements (essential, potentially toxic, and toxic elements), such as aluminum (Al), barium (Ba), beryllium (Be), bismuth (Bi), cadmium (Cd), cobalt (Co), chromium (Cr), copper (Cu), iron (Fe), gallium (Ga), manganese (Mn), molybdenum (Mo), nickel (Ni), lead (Pb), strontium (Sr), thallium (Tl), and zinc (Zn), in the blood serum and blood clot of grass carp (*Ctenopharyngodon idella*), as well as in water and sediment samples. The next objective was to assess the health status of the fish and evaluate biochemical parameters such as sodium (Na), potassium (K), chloride (Cl), calcium (Ca), phosphorus (P), magnesium (Mg), urea, total proteins (TP), glucose (Glu), aspartate aminotransferase (AST), alanine aminotransferase (ALT), alkaline phosphatase (ALP), gamma glutamyl transferase (GGT), cholesterol (Chol), triglycerides (TAG), and oxidative status/stress markers like reactive oxygen species (ROS) production, total antioxidant capacity (TAC), protein carbonyls (PC), lipid peroxidation (LPO), glutathione peroxidase (GPx) activity, total bilirubin (Bili), uric acid (UA), and creatinine (Crea). The final goal of this work was to clarify the possible connections between the studied biomarkers and concentrations of trace elements. Our study can provide information about the impact of these pollutants on a complex of biochemical parameters, oxidative stress markers, and potential health consequences in natural conditions.

## Material and methods

### Fish and experimental design

**Fig. 1:**
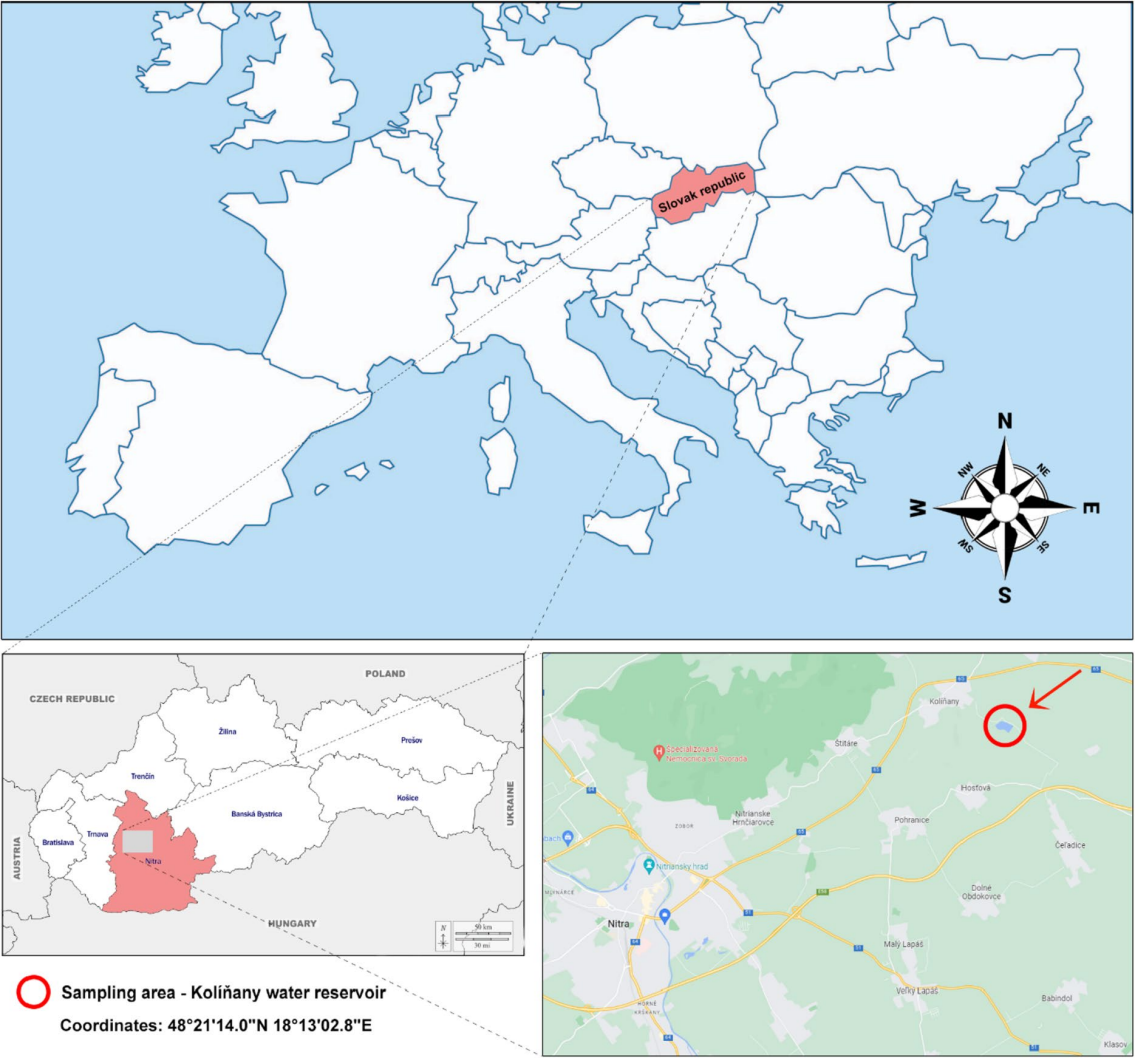
Sampling area—The University experimental fishpond Kolíňany (West Slovak Lowland—Slovak Republic)

This work was realized during the summer and autumn seasons. Adult (4-year-old) grass carps were harvested from a university experimental pond in Kolíňany—West Slovak Lowland—Slovak Republic (48°21′14.0″N 18°13′02.8″E) (Fig. 1). The location selected for this study is characterized by intensive agricultural activity; the university experimental fishpond also serves as a catchment area for the wastewater treatment plant. Fish stocking was realized one year before the first sampling. The freshwater fish (*Ctenopharyngodon idella*) were caught using a seine net, according to our previous study (Kovacik et al., 2019). In total, 36 fish were collected. After catching, the fish were promptly transferred to the laboratory in polyethylene bags within 20 min for the collection of blood samples. Blood collection was realized following a standard ichthyology assessment [total length (TL), standard length (SL), and weight measurements] (Table 1). The animals were carefully managed by a capable individual in compliance with the regulations specified in the national law. All experiments were conducted in adherence to the guidelines set forth by the Ethics Committee for Protection of Animals Used for Scientific and Teaching Purposes of the Slovak University of Agriculture in Nitra. The Ethics Committee of the Slovak University of Agriculture in Nitra (SUA in Nitra) for the protection of animals used for scientific and educational purpose confirms that no special ethical approval was required for this type of experiment. During biological material collection, sampling of water and sediment from the experimental pond was also realized. To facilitate comparison with other authors who provided the total length (TL) of fish, the transformation equation is presented as follows: TL = 9.56889 + 1.15693*SL (r = 0.9932; r^2^ = 98.64%).

**Table 1:** Standard ichthyology evaluation of analysed fish (*Ctenopharyngodon idella*)

	n	Age (year)	SL (mm)		BW (g)	
			Mean ± SD	Min–Max	Mean ± SD	Min–Max
Summer season	18	4	431.7 ± 38.9	355.0–510.0	1383.0 ± 364.9	682.0–2088.0
Autumn season	18	4	414.7 ± 24.5	365.0–450.0	1243.5 ± 244.0	634.0–1617.0
Total	36		422.9 ± 33.1	355.0–510.0	1311.4 ± 312.5	634.0–2088.0

SL—standard length, BW—body weight, SD—standard deviation, n—number of individuals, Min -minimum and Max – maximum.

### Sample collection

The sampling of fish (n = 36) was realized in the summer (n = 18) and autumn (n = 18) seasons from adult fish (4-year-olds). Blood samples were collected from the *aorta ventralis* of each individual and placed into three collection tubes. The samples were allowed to coagulate and subsequently centrifuged for 20 min at 1006 × *g* and 20 °C. Collected blood serum and blood clot samples were stored at -20 °C until analyses at the Institute of Applied Biology (SUA in Nitra, Slovakia).

Water and sediment samples were collected during fish harvesting. Sterile bottles were used for water sampling and were rinsed with pond water at the sampling site. About 200 mL of water was collected by immersing it to a depth of about 25 cm. Sediment samples were collected using a Snapper sediment sampler (about 150 g). All samples were collected from the inflow, middle, and dam parts of the pond. In each parts 4 samples of water and 4 samples of sediment were collected, covering litoral and pelagic zones. We took one extra sample of water from the inflow part of the pond due to the stronger water flow in this part. Samples were collected in duplicate and stored in a portable refrigerator at a temperature of 4 °C.

### Measurements of serum biochemistry parameters and oxidative status markers

Sodium (Na), potassium (K), and chloride (Cl) ions were measured using the EasyLite analyser (Medica, Bedford, MA, USA) provided with an ion-selective electrode. Calcium (Ca), phosphorus (P), magnesium (Mg), urea, total proteins (TP), glucose (Glu), aspartate aminotransferase (AST), alanine aminotransferase (ALT), alkaline phosphatase (ALP), gamma glutamyl transferase (GGT), total bilirubin (Bili), cholesterol (Chol), triglycerides (TAG), uric acid (UA), and creatinine (Crea) were analysed using standard commercial kits from DiaSys (Diagnostic Systems GmbH, Holzheim, Germany) on the semi-automated clinical chemistry analyser Randox RX Monza (Randox Laboratories, Crumlin, UK) (Kovacik et al., 2017, 2019; Massanyi et al., 2014). Total bilirubin, creatinine, and uric acid values were recalculated to grams of total protein; thus, the outcomes are presented in terms of μmol/g total protein (Bili and Crea) and mg/g total protein (UA).

Reactive oxygen species (ROS) production was quantified by the chemiluminescence assay according to Kashou et al. (2013) and optimized for our laboratory conditions as in our previous studies (Kovacik et al., 2018, 2019; Tvrdá et al., 2016a, 2016b; Tvrdá et al., 2016a, 2016b). The results are expressed as relative light units (RLU)/s/g total protein.

Total antioxidant capacity (TAC) was assessed using the improved chemiluminescence antioxidant assay, which utilizes the horseradish peroxidase conjugate and luminol according to Muller et al. (2013) and is optimized for our laboratory conditions as in our previous studies (Kovacik et al., 2018, 2019). The outcomes are presented in terms of μmol of Trolox Eq./g of total protein.

The quantification of the carbonyl group was accomplished using the conventional 2,4-dinitrophenylhydrazine (DNPH) method. The expression of protein carbonyls is given in units of nmol per gram of total protein (Kovacik et al., 2019; Tvrda et al., 2017; Weber et al., 2015).

Lipid peroxidation (LPO) was measured by the quantification of malondialdehyde (MDA) production with the help of the TBARS assay, according to Tvrdá et al., 2016a, 2016b; Tvrdá et al., (2016a, 2016b). MDA concentrations are expressed as μmol per gram of total protein (Kovacik et al., 2019).

Glutathione peroxidase (GPx) activity was assessed using the Randox commercial kits (Randox Laboratories, Crumlin, Great Britain) and the semiautomated analyser Randox RX Monza (Randox Laboratories, Crumlin, UK). The findings are reported in units per gram of total protein (Tvrdá et al., 2016a, 2016b).

### Determination of trace elements

The levels of selected trace elements (Al, Ba, Be, Bi, Cd, Co, Cr, Cu, Fe, Ga, Mn, Mo, Ni, Pb, Sr, Tl, and Zn) in various matrices (blood serum, blood clot, water, and sediment) were analysed using inductively coupled plasma optical emission spectrometry (ICP Thermo ICAP 7000 Dual (Thermo Fisher Scientific, Waltham, Massachusetts, USA)).

### Pre-analytical procedure for ICP OES

Sample (blood serum and blood clot, about 500 mg) preparation for ICP OES analysis was carried out by closed-vessel microwave acid digestion with the high-performance microwave digestion system Ethos UP (Milestone Srl, Sorisole, BG, Italy) according to our previous studies (Kovacik et al., 2018, 2019). The same conditions for mineralization (amount of chemicals, time, temperature, and power) were used for sediment samples (about 500 mg). For this procedure, all the chemicals used had high purity grade to minimize contamination.

Water samples were measured directly after filtration using Whatman® qualitative filter paper, Grade 595 (basis weight 68 g/m^2^, thickness 150 µm, pore size 4–7 µm).

### ICP OES analysis

Elemental determination of selected elements (Al, Ba, Be, Bi, Cd, Co, Cr, Cu, Fe, Ga, Mn, Mo, Ni, Pb, Sr, Tl, and Zn) was carried out using the spectrophotometer ICP Thermo ICAP 7000 Dual (Thermo Fisher Scientific, Waltham, Massachusetts, USA). A calibration curve was created using different dilutions (1:5, 1:25, 1:100, and 1:500) of Multielement Standard Solution V for ICP (Sigma-Aldrich Production GmbH, Switzerland) (Kovacik et al., 2018, 2019). The instrumental parameters of the determination with which it was carried out are listed in Table 2. Detection limits (LOD) of elements for blood serum (mg/kg), blood clot (mg/kg), sediment (mg/kg), and pond water (mg/L) were Al 0.0002; Ba 0.004; Be 0.001; Bi 0.020; Cd 0.0004; Co 0.003; Cr 0.017; Cu 0.003; Fe 0.002; Ga 0.026; Mn 0.0004; Ni 0.0002; Pb 0.009; Sr 0.002; Tl 0.005; and Zn 0.0006. Quantification limits (LOQ) of elements for blood serum (mg/kg), blood clot (mg/kg), sediment (mg/kg), and pond water (mg/L) were Al 0.001; Ba 0.013; Be 0.002; Bi 0.064; Cd 0.001; Co 0.008; Cr 0.055; Cu 0.011; Fe 0.052; Ga 0.085; Mn 0.013; Ni 0.001; Pb 0.032; Sr 0.007; Tl 0.016; and Zn 0.002. In all analyses, we used certified reference material for verification (CRM-ERM CE278K, Sigma-Aldrich Production GmbH, Switzerland).

**Table 2:** Operating parameters for the determination of trace elements by ICP OES

Method parameters	
RF power (kW)	1.15
Plasma flow (L/min)	normal
Auxiliary flow (L/min)	0.50
Nebulizer flow (L/min)	0.40
Coolant Gas Flow (L/min)	12
Sample uptake delay (s)	30
Pump rate (rpm)	50
Rinse time (s)	30
Element (nm)	Al (I) 167.079, Ba (II) 455.403, Be (II) 313.042, Bi (I) 223.061, Cd (II) 214.438, Co (II) 238.892, Cr (II) 284.325, Cu (I) 324.754, Fe (II) 238.204, Ga (I) 294.364, Mn (II) 257.610, Mo (II) 204.598, Ni (II) 221.647, Pb (II) 220.353, Sr (II) 421.552, Tl (II) 190.856, Zn (II) 206.200

(I) – atomic line; (II) – ionic line.

### Statistics

Before statistical analyses, we subjected the obtained data to normality tests using Kolmogorov–Smirnov. Subsequently, we evaluated the significance of the differences between the experimental groups divided based on the season in different matrices (blood serum or blood clot) using an unpaired t-test. Results are presented as the mean ± SD (standard deviation). Spearman *R* correlations were used to determine mutual associations between trace elements and biochemical markers. We also subjected the possible associations between selected trace elements and oxidative status markers to correlation analysis. The statistically significant correlations of this relationship were evaluated using regression analysis. A *p* value below 0.05 was set as statistically significant. STATGRAPHICS Centurion (© StatPoint Technologies, Inc., USA), GraphPad Prism 6.01 (GraphPad Software Incorporated, San Diego, California, USA), and Minitab® statistical software (Minitab Ltd., Coventry, UK) were used for statistical evaluation.

## Results

In the present study, we used four matrices (blood serum, blood clot, water from the sampling area, and sediment from the sampling area) to quantify selected trace elements. Minimum and maximum concentrations are presented in Table 3. The most accumulated elements were Zn in the blood serum, Fe in the blood clot, Sr in the water, and Al in the sediment. The general trend of decreasing levels of trace elements in the blood serum samples was as follows: Zn ˃ Fe ˃ Sr ˃ Ba ˃ Ni ˃ Al ˃ Cu ˃ Be ˃ Co; < LOQ (below limit of quantification): Bi, Cd, Cr, Ga, Mn, Mo, Pb, and Tl; and in case of the blood clot, the scheme was as follows: Fe ˃ Zn ˃ Sr ˃ Al ˃ Ni ˃ Ba ˃ Cu ˃ Be ˃ Co ˃ Mn; < LOQ (below limit of quantification): Bi, Cd, Cr, Ga, Mo, Pb, and Tl. In the water samples, almost all monitored elements were below the limit of quantification, except for Al, Ba, Cu, and Sr. In the sediment samples, the values of the monitored elements were generally the highest, and the scheme was as follows: Al ˃ Fe ˃ Mn ˃ Ba ˃ Sr ˃ Zn ˃ Cr ˃ Cu ˃ Ni ˃ Pb ˃ Co ˃ Be ˃ Cd; < LOQ (below limit of quantification): Bi, Ga, Mo, and Tl.

**Table 3:** Ranges of trace elements concentration in the matrices blood serum and blood clot of *Ctenopharyngodon Idella*, and in the water and sediment from the sampling area

Element	Blood serum (n = 36)	Blood clot (n = 36)	Pond water (n = 26)	Pond sediment (n = 24)
	(mg/kg, ww)	(mg/kg, ww)	(mg/L, ww)	(mg/kg, ww)
Al	0.282–1.894	0.560–4.149	^2^ < LOQ-0.010	22 378.897–75 922.810
Ba	^2^ < LOQ-2.722	0.386–1.958	0.030–0.068	119.130–318.419
Be	0.274–1.192	0.126–0.529	< LOQ	0.670–1.599
Bi	< LOQ	< LOQ	< LOQ	< LOQ
Cd	< LOQ	< LOQ	< LOQ	0.144–0.695
Co	^18^ < LOQ-0.418	^1^ < LOQ-0.243	< LOQ	9.231–16.578
Cr	< LOQ	< LOQ	< LOQ	16.892–41.938
Cu	^1^ < LOQ-2.273	0.215–0.561	^2^ < LOQ-0.096	7.342–78.113
Fe	1.526–12.224	216.879–603.928	< LOQ	17 502.627–36 084.599
Ga	< LOQ	< LOQ	< LOQ	< LOQ
Mn	< LOQ	^1^ < LOQ-0.146	< LOQ	273.805–536.711
Mo	< LOQ	< LOQ	< LOQ	< LOQ
Ni	^1^ < LOQ-2.168	^1^ < LOQ-5.339	< LOQ	16.178–55.721
Pb	< LOQ	< LOQ	< LOQ	4.115–30.542
Sr	0.323–5.060	0.725–3.498	^2^ < LOQ-0.560	77.576–162.202
Tl	< LOQ	< LOQ	< LOQ	< LOQ
Zn	4.650–12.269	5.103–10.516	< LOQ	20.529–352.891

< LOQ = below limit of quantification. The number indicates how many of the samples were below the quantification limit.

**Fig. 2:**
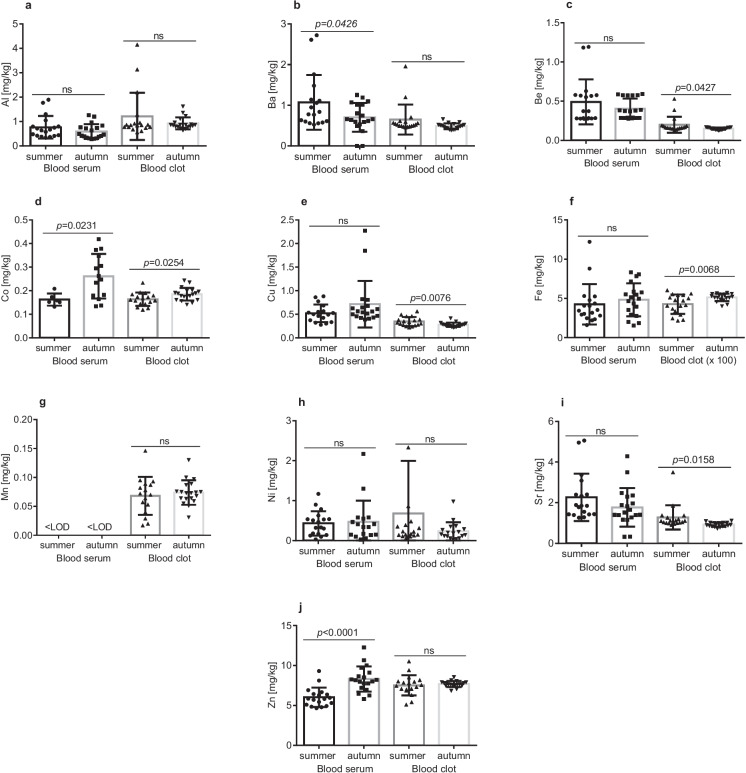
Trace elements concentrations in blood serum and blood clot of *Ctenopharyngodon idella* (n = 18 in each season) from the sampling area in different seasons (summer and autumn season). Values are presented as mean ± SD (standard deviation); ns – not significant

The mean seasonal levels of trace elements in the blood serum and blood clot are presented in Fig. 2 (mean ± SD). Significant differences between the seasons were detected for concentrations of Ba, Co, and Zn in the blood serum and of Be, Co, Cu, Fe, and Sr in the blood clot. Overall, Ba, Be, Cu, Ni, and Sr content was higher in the blood serum, while Al, Co, Fe, Mn, and Zn content was higher in the blood clot.

**Fig. 3:**
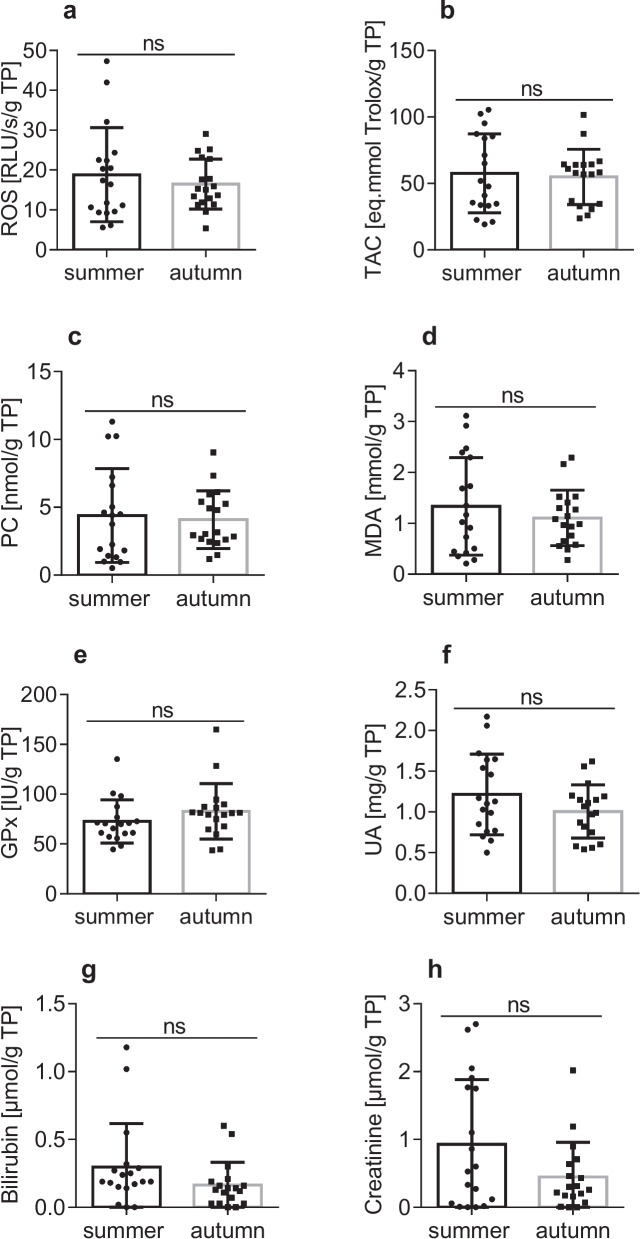
Oxidative stress markers measured in blood serum of *Ctenopharyngodon idella* in different seasons. ns—not significant. GPx – glutathione peroxidase; PC – protein carbonyls; ROS – reactive oxygen species; TAC—total antioxidant capacity, MDA—malondialdehyde production; UA – uric acid

Mean concentrations of blood biochemical parameters are reported in Table 4. Mg, Na, Cl, TP, glucose, AST, ALT, cholesterol, and triglyceride concentrations were significantly higher in the autumn season (*p* < 0.05). K level was significantly higher in the summer season (*p* < 0.05). Non-significant differences were found in the cases of Ca, P, urea, and ALP levels. The mean (± SD) concentrations of selected biomarkers (ROS, TAC, GPx, PC, MDA, UA, Bili, and Crea) among seasons are presented in Fig. 3. We did not record significant differences in this group of evaluated parameters.

**Table 4:** Blood biochemical parameters of *Ctenopharyngodon idella* in different seasons. Data are presented as mean values, standard deviation, and coefficient of variation

Parameter	Season						*p* value
	Summer			Autumn			
	Mean	SD	CV%	Mean	SD	CV%	
Ca (mmol/L)	2.94	0.35	11.82	3.09	0.44	14.12	ns
P (mmol/L)	4.49	1.36	30.35	5.23	0.94	18.13	ns
Mg (mmol/L)	**2.35**	0.24	10.13	**3.09**	0.36	11.71	*0.0001*
Na (mmol/L)	**124.75**	5.37	4.31	**137.51**	3.52	2.56	*0.0001*
K (mmol/L)	**2.72**	2.25	82.49	**1.04**	0.68	65.89	*0.0037*
Cl (mmol/L)	**100.44**	5.82	5.79	**109.08**	3.41	3.13	*0.0001*
Total Proteins (g/L)	**21.71**	3.66	16.87	**30.20**	4.03	13.34	*0.0001*
Glucose (mmol/L)	**6.67**	1.89	28.45	**10.08**	4.19	41.62	*0.0003*
Urea (mmol/L)	1.18	0.77	65.28	1.11	0.54	48.21	ns
AST (µkat/L)	**1.05**	1.28	122.13	**3.37**	1.87	55.35	*0.0001*
ALT (µkat/L)	**0.15**	0.06	40.77	**0.30**	0.11	37.49	*0.0001*
ALP (µkat/L)	1.82	1.18	65.00	1.18	0.82	69.04	ns
GGT (µkat/L)	**0.08**	0.07	114.36	**0.02**	0.03	174.59	*0.0091*
Cholesterol (mmol/L)	**3.73**	0.71	19.05	**6.62**	1.33	20.03	*0.0001*
Triglycerides (mmol/L)	**1.98**	0.73	36.91	**3.26**	0.76	23.24	*0.0001*

Bold values are statistically significant; SD—standard deviation; CV%—coefficient of variation; ns—not significant.

The Spearman *R* correlation coefficients and linear or non-linear regression were used to evaluate direct relationships between trace elements and selected blood biomarkers. Statistically significant correlations between trace elements and serum biochemistry parameters (divided according to the season and matrices) are presented in Table 5.

**Table 5:** Statistically significant correlations between concentrations of trace element in the blood serum/clot and biochemical parameters in different seasons

Summer season				Autumn season			
Biochemical parameter	Trace element (matrice)	Spearman *R*	*p* value	Biochemical parameter	Trace element (matrice)	Spearman *R*	*p* value
Na	Al serum	-0.5534	0.0269	Cl	Be clot	-0.4844	0.0399
Na	Ba serum	-0.5117	0.0407	Glucose	Fe serum	0.5845	0.0131
Na	Fe serum	-0.6614	0.0082	Glucose	Al clot	0.4660	0.0480
Na	Ni serum	-0.5215	0.0370	Glucose	Ba clot	0.5171	0.0282
Na	Sr clot	-0.5055	0.0432	Glucose	Be clot	0.5073	0.0314
K	Al serum	0.5490	0.0281	Glucose	Cu clot	-0.5494	0.0198
K	Ba serum	0.5417	0.0303	P	Zn serum	0.5125	0.0297
K	Ni serum	0.6446	0.0099	P	Ni clot	0.5108	0.0302
K	Sr serum	0.5383	0.0313	Mg	Zn serum	0.4941	0.0361
K	Cu clot	0.5270	0.0350	Mg	Cu clot	-0.5731	0.0150
K	Fe clot	-0.4975	0.0466	Ca	Zn serum	0.6462	0.0061
K	Sr clot	0.5809	0.0202	AST	Fe serum	0.5298	0.0246
P	Al serum	0.5700	0.0188	AST	Zn serum	0.6719	0.0044
P	Ba serum	0.5379	0.0266	AST	Cu clot	-0.5263	0.0256
P	Cu serum	0.6254	0.0124	ALT	Zn serum	0.6982	0.0031
P	Ni serum	0.7269	0.0027	ALT	Be clot	0.5158	0.0286
P	Sr serum	0.4897	0.0435	ALT	Cu clot	-0.5018	0.0333
P	Zn serum	0.6082	0.0122	ALP	Al clot	-0.4721	0.0452
P	Ba clot	0.5248	0.0305	TAG	Be clot	-0.5272	0.0253
Mg	Zn serum	0.5021	0.0384	Chol	Zn serum	0.8070	0.0006
AST	Al serum	0.5377	0.0266	TP	Zn serum	0.8561	0.0003
AST	Ba serum	0.6574	0.0067	Urea	Fe serum	0.5125	0.0297
AST	Be serum	0.6160	0.0111	Urea	Zn serum	0.5151	0.0288
AST	Fe serum	0.6656	0.0061	Urea	Be clot	0.4712	0.0456
AST	Sr serum	0.7135	0.0033	Urea	Cu clot	-0.5406	0.0218
AST	Ni clot	0.5810	0.0166				
ALT	Al serum	0.5666	0.0195				
ALT	Fe serum	0.5728	0.0182				
ALT	Ni serum	0.5418	0.0255				
ALT	Sr serum	0.5576	0.0215				
ALT	Sr clot	0.6326	0.0091				
ALP	Fe serum	0.5769	0.0174				
ALP	Sr serum	0.5638	0.0201				
TAG	Fe serum	0.5666	0.0195				
TAG	Sr clot	0.4964	0.0407				
GGT	Al clot	0.5298	0.0289				
GGT	Be clot	0.5437	0.0250				
TP	Ba serum	0.5253	0.0303				
TP	Cu serum	0.5098	0.0414				
TP	Fe serum	0.5748	0.0178				
TP	Sr serum	0.5421	0.0254				
TP	Zn serum	0.5666	0.0195				
Urea	Ni serum	0.5501	0.0233				
Urea	Ba clot	0.6195	0.0106				
Urea	Be clot	0.5503	0.0233				
Urea	Sr clot	0.4923	0.0424				

In the summer season, negative statistically significant correlations between the serum Al, Ba, Fe, Ni, clot Sr concentrations, and Na were found. Positive (statistically significant) correlations were found between serum Al, Ba, Ni, Sr, and clot Cu, Sr vs. K. P positively correlated with serum Al, Ba, Cu, Ni, Sr, Zn, and clot Ba. Zn in the serum was positively correlated with Mg. Enzymes such as AST positively correlated with serum Al, Ba, Be, Fe, Sr, and clot Ni, and ALT positively correlated with serum Al, Fe, Ni, Sr, and clot Sr. Concentrations of ALP were positively correlated with serum Fe and Sr. Fe in the serum and Sr in the blood clot were positively correlated with TAG levels. Al and Be in the blood clot positively correlated with GGT levels. TP was positively correlated with the serum elements (Ba, Cu, Fe, Sr, and Zn). Positive statistically significant correlations between the serum Ni, clot Ba, Be, and Sr concentrations vs. urea were confirmed as well.

The correlation analysis in the autumn season showed a significant negative association between Cl and Be (clot). Cu in the blood clot is negatively correlated with glucose, Mg, AST, ALT, and urea. Glucose levels were also positively correlated with Fe in the serum and Al, Ba, and Be in the blood clot. Positive (statistically significant) correlations were found between serum Zn vs. P, Mg, Ca, AST, ALT, cholesterol, TP, and urea. Be in the blood clot significantly correlated with ALT, TAG, and urea. Serum Fe was also positively correlated with AST and urea levels. Positive and significant relationships were observed between Ni (clot) and P and Al (clot) and ALP.

**Fig. 4:**
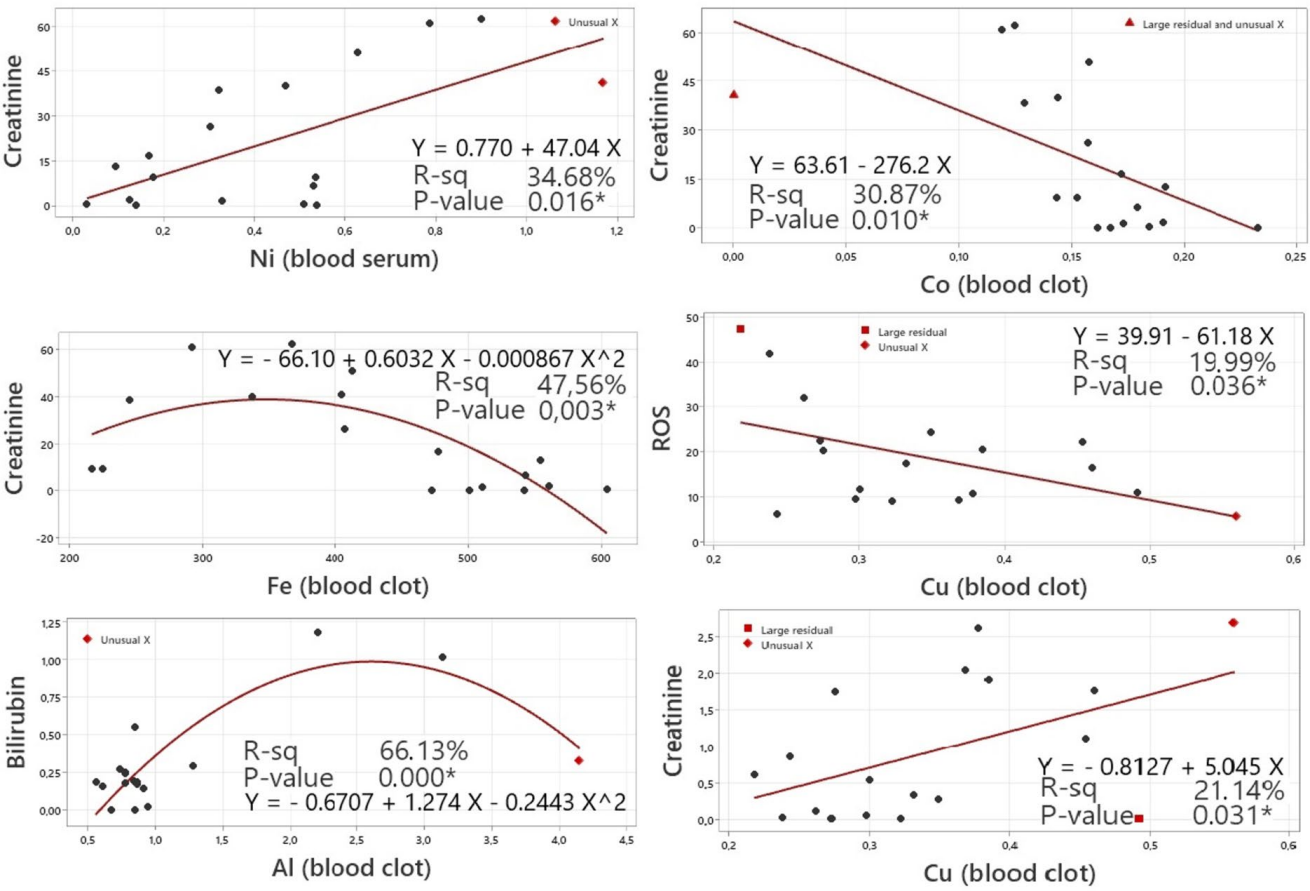
Dependency between the trace elements concentration and oxidative stress markers with significant correlation coefficient in summer season. The straight/curve line represents the best fit line obtained by linear or non-linear regression

Linear and non-linear regression analysis was used to explain the dependencies between trace element concentrations and oxidative status markers. The results of the regression analysis for the monitored parameters in the summer season are listed in Fig. 4. Strong statistically significant correlations were detected between Crea and serum Ni (0.612), as well as Fe (-0.576), Co (-0.609), and Cu (0.508) in the blood clot. A positive statistically significant correlation was observed between bilirubin and Al in the blood clot (0.574). A moderately negative (-0.497) significant association was detected between ROS and Cu in the blood clot.

**Fig. 5:**
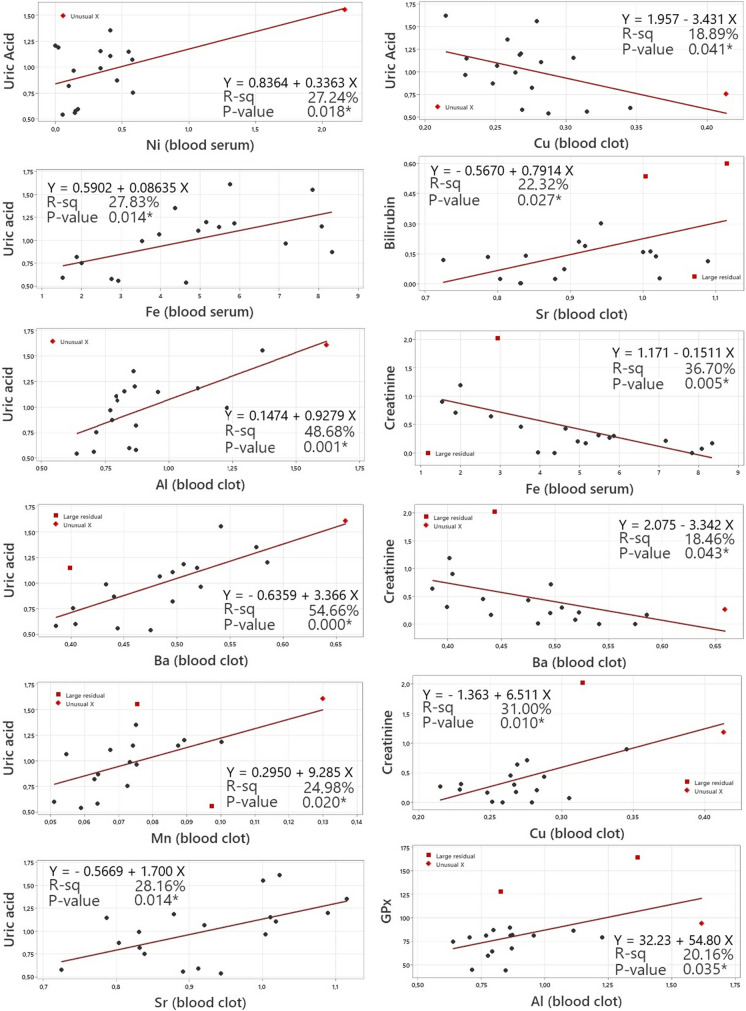
Dependency between the trace elements concentration and oxidative stress markers with significant correlations in autumn season. The straight line represents the best fit line obtained by linear regression

Dependencies between trace element concentrations and RedOx status parameters in the autumn season are presented in Fig. 5. Strong or moderate statistically significant correlations were detected between UA and Ni (0.550), Fe (0.565) in the serum, and Al (0.719), Ba (0.754), Cu (-0.469), Mn (0.472), and Sr (0.563) in the blood clot. A moderately positive and significant association was detected between bilirubin and Sr in the blood clot (0.464), as well as GPx and Al in the blood clot (0.505). Creatinine levels were significantly associated with serum Fe (-0.637), Ba (-0.546), and Cu (0.613) in the blood clot.

## Discussion

Due to the persistent pollution of the environment with various contaminants, it is increasingly important to monitor the concentration of dangerous substances in living and non-living nature. There are numerous studies based on biomonitoring of the content of trace elements in various fish matrices (blood, muscle, gills, liver, kidney, or spermatozoa) (Ansel & Benamar, 2018; Hansson et al., 2020; Karjalainen et al., 2020; Malik et al., 2010; Monferrán et al., 2016; Rajeshkumar et al., 2018). On the other hand, there is a relatively small amount or even a lack of studies describing possible associations of pollutants with various animal blood biomarkers in natural conditions. As such, the present study is dealing with the monitoring of trace element content in the blood serum, blood clots of grass carp, and in the pond water, and sediment. A substantial part of our study looks for relevant associations between the animal organism and trace elements, health status parameters, and markers of oxidative stress. For this type of study, we consider freshwater animals to be an ideal biological material, as they are in constant contact with their surrounding environment.

### Monitored trace elements in different matrices

We monitored the content of various microelements over two seasons. Samples were not collected in the winter due to the hibernation of the fish, while we let the fish spawn in the university's experimental pond in the springtime. For these reasons, fishing took place from summer to autumn. We quantified Al, Ba, Cu, and Sr in water samples. Contamination of surface waters occurs primarily from natural sources, but anthropogenic sources of pollution (combustion processes, industrial activity, intensive agriculture, waste disposal, mining, and smelting) are the main reason for increased levels of toxic elements in the environment (Kolarova & Napiórkowski, 2021). The collected sediments from the selected site contained all monitored elements in measurable concentrations except for Bi, Ga, Mo, and Tl. In general, sediments form an ideal reservoir for the deposition of toxic elements, which is subsequently manifested in the accumulation of these substances in living organisms (Emenike et al., 2022). The basic determination of the content of microelements in blood serum may be counterploted with our previous study carried out on *Cyprinus carpio* (Kovacik et al., 2019). Similarly to our collected data, Zn and Fe were among the most accumulated, which is logical due to their general biological relevance and occurrence in the animal organism. A positive finding of this study lies in the undetected levels of traditional toxic elements (Cd and Pb) in the blood serum and blood clot of grass carp in comparison to our previous study conducted on the common carp. At the same time, concentrations of other elements such as Bi, Cr, Ga, Mo, and Tl were below the quantification limits. Our data confirm a seasonal impact on the contents of Ba, Be, Cu, and Sr (increased in the summer season) as well as Co, Fe, and Zn (increased in the autumn season). Currently, there is a lack of studies monitoring the content of trace elements in the blood of freshwater animals. Most biomonitoring studies are concerned with the metal content in tissues (especially in muscle) due to their potential health risks for consumers (Bosch et al., 2016; Dhar et al., 2023; Varol et al., 2022) without further biological relevance or physiological and/or pathological interactions. One of the few studies published to this date describes seasonal variations of selected elements in the muscle, gill, and liver of the freshwater fish *Esox lucius*. While the tendencies were different in comparison to our results, if we focus on the liver, the highest accumulation of most metals (Al, Ba, Cr, Cu, Fe, Pb, and Zn) was recorded in the spring and summer with a gradual decrease in autumn and winter (Erdoğan et al., 2021). Nevertheless, focusing not only on the tissues but also on the blood of fish, the content of elements and biomarkers can reveal various physiological indications and pathological changes (Emenike et al., 2022; Shahjahan et al., 2022a, 2022b).

### Trace elements and serum biochemistry parameters

Previous studies primarily describe the accumulation of trace elements in water or in tissues associated with the haematological and biochemical functions of the organism. Khan et al. (2020) confirmed a direct association between increased liver enzymes (AST, ALT, ALP, and CK) and selected haematological parameters and a rise in the content of Cr, Mn, Fe, Ni, Cu, and Zn in the muscle, liver, and kidney of *Oreochromis niloticus* (Nile tilapia). Tlenshieva et al. (2022) observed genotoxic effects and histopathological changes in the liver of grass carp (*Ctenopharyngodon idella*) in metal (Pb, Co, Mg, Cd, Cu, Zn, and Fe) contaminated locations. The authors suggested a direct association of these changes (especially gill lesions) with the level of water contamination. In our study, we may discuss the associations of Al, Ba, Fe, Ni, Sr, Cu, or Zn with the mineral profile of the blood (Na, K, Mg, and P) in the summer season, as well as a possible response of liver enzymes (AST, ALT, ALP, and GGT). We also recorded an increased content of AST, ALT, cholesterol, and triglycerides in the autumn season, confirming association with concentrations of Fe, Zn, Cu, Be, and Al. The Zn content was significantly higher in the autumn period, while at the same time, its associations with increased concentrations of TP and urea were observed. Increased liver enzymes (ALT and AST) and cholesterol due to higher Cu levels were also noted by Öner et al. (2008) in *Oreochromis niloticus*. The effect of Zn on biochemical markers was not confirmed in their study, except for the association with increased cholesterol in the blood. In our already mentioned previous study carried out on common carp (Kovacik et al., 2019), several associations between blood minerals (Ca, Mg, Na, K, Cl) and trace elements were confirmed in the spring (Sr, Zn, Cd, Fe, Mn) and summer (Zn, Cr, Cu, Fe, Hg, Ni, As) seasons, followed by a negative correlation of Ni with the content of TP, Chol, and TG, or by a positive association of Cu vs. Chol or TG. According to Fırat and Kargın (2010), a single and combined metals exposure, represented by 5.0 mg/L Zn, 1.0 mg/L Cd, and 5.0 mg/L Zn + 1.0 mg/L Cd mixtures, was tested on a freshwater fish, *Oreochromis niloticus*, for 7 and 14 days, leading to an increase in TP, ALT, AST, albumin, transferrin, ceruloplasmin, cortisol, and glucose, and a decrease in cholesterol concentration. Banday et al. (2019) analysed the effects of trace elements on the health status of freshwater *Clarias gariepinus*. The authors obtained the fish from a river contaminated by several sources of contamination (power plants, agricultural waste, and domestic waste). The study monitored the content of Cd, Ni, Cu, and Cr in the integument, liver, blood, spleen, head-kidney, and thymus, where the highest content of the elements was found in the liver and blood. The authors noted changes in serum chemistry, haematology, hormonal levels, severe pathological alterations in immune organs, or elevations in hepatic and renal enzymes. In the meantime, Abdel-Tawwab et al. (2016) reported that exposure to sublethal Zn concentrations significantly increased glucose, AST, ALT, creatinine, cortisol, proteins, and lipid levels in *Oreochromis niloticus* (L.). On the other hand, Atli et al. (2015) recorded a decrease in the activity of ALP, cholesterol, and triglyceride levels due to acute (10 μM, 2 d) and chronic (20 μM, 20 d) exposure to Cd and Pb. Furthermore, the authors noted an increase in the concentration of glucose levels, partially due to the impact of Cd. Nevertheless, the results of serum chemical markers did not seem to be consistent enough. This may hinder the comparison of studies, especially because of the number of freshwater and marine species used in studies. Nevertheless, we consider blood biochemical markers to be a very important diagnostic tool for the early detection of various physiological changes and diseases caused by contaminants (Shahjahan et al., 2022a, 2022b).

### Trace elements and oxidative status markers

RedOx (oxidative status/stress) markers are nowadays widely used to measure the extent of toxicity caused by environmental pollutants such as trace elements (Hinojosa-Garro et al., 2020; Kovacik et al., 2019; Shahjahan et al., 2022a, 2022b), endocrine disruptors (Jambor et al., 2018; Lee et al., 2019), pesticides (Clasen et al., 2018; Shahjahan et al., 2022a, 2022b), or microplastics (Alomar et al., 2017; Capó et al., 2022). This interpretation is based on the fluctuations of several markers, such as ROS, TAS, and enzymatic and non-enzymatic antioxidant defence systems. In our study, we monitored an entire complex of markers. As the main parameters, we evaluated ROS and TAC, supported by enzymatic (GPx) and non-enzymatic endogenous markers of oxidative stress (PC, MDA, Bili, UA, and Crea). Based on our findings, we may deduce a slight decrease in all monitored markers in the autumn season except for GPx, but without statistical evidence, which may indicate a relative resilience of the RedOx markers towards the external environment. Some interesting insights were provided by the correlation analysis evaluated by linear and/or non-linear regression, where the ROS levels were affected by the Cu content in the blood clot in the summer season. In general, several studies describe the redox potential of copper, which may be divided into two groups. The first one talks about the lack of copper in the organism, as a result of which oxidative stress can occur, whereas the second one describes increased concentrations of copper, followed by higher levels of ROS as well as changes in the enzymatic antioxidant system in aquatic animals (Baldissera et al., 2020; Do Carmo et al., 2018; El-Sharawy et al., 2022; Jiang et al., 2011; Lushchak, 2016; Machado et al., 2013; Nunes et al., 2015). Lakra et al. (2022) tested the overall health status of catfish (*Clarias batrachus*) reared in wastewater from a coal mine. When compared to the control group, the authors recorded increased concentrations of metals (Fe, Zn, Cu, Mn, Ni, Cd, Pb, and Cr) in all tested tissues (skin, air-breathing organ, gills, liver, kidney, and brain). Subsequent analyses revealed a negative effect on the content of all monitored biomarkers (glucose, glycogen, lipid, and protein), as well as on enzymatic parameters (AST, ALT, and ALP) and markers of oxidative stress (superoxide dismutase, catalase, and lipid peroxidation). Subsequently, after transferring the experimental fish to tap water, they observed a decrease in the activity of antioxidant enzymes as well as other health markers. In a different study monitoring *Astyanax aeneus* as a bioindicator of pollution exposed to the natural environment, in addition to the content of trace elements (Zn, Cd, Pb, Cu, Cr, Fe, Al, Mn, Hg, As, and V), the authors also observed associations with selected biomarkers (Hinojosa-Garro et al., 2020). Furthermore, the study revealed increased activities of glutathione S-transferase, metallothionein, catalase, and TBARs in selected locations, which could be directly related to the increased metal content.

In our study, we also noted associations between Ni, Co, Fe, and Cu and creatinine content, while Al was associated with bilirubin in the summer season. On the other hand, correlations amongst Ni, Fe, Al, Ba, Mn, Sr, and Cu with UA concentrations were confirmed; Sr was correlated with bilirubin; Fe, Ba, and Cu with creatinine; and Al with GPx in the autumn season. Seasonal variations of metal contents (Cr, Cd, Cu, Zn, Mn, and Fe) in water and their subsequent association with cichlid blood biomarkers were monitored by Ruas et al. (2008). In the contaminated site, the authors recorded increased levels of Cr, Cu, Zn, and Fe in the spring when compared to the autumn. These increased concentrations were subsequently associated with increased lipid peroxidation as well as SOD activity, accompanied by a partial decrease in the enzymatic activity of catalase and GPx and a reduction of glutathione. Giarratano et al. (2016) confirmed seasonal variations of Cd, Cu, Cr, Pb, Zn, Ni, and Al in tissues collected from the crab *Neohelice granulata*. Furthermore, they also found associations between these elements and markers of oxidative stress, specifically through the induction of glutathione in connection with Fe content or a significant effect of Al on MDA (lipid peroxidation).

## Conclusion

A positive finding of the research task is the very low concentration of classical toxicants under the limits of detection (Cd, Pb, and Tl) in blood serum/clot and water samples. All monitored microelements except Bi, Ga, Mo, and Tl were present in the sediment. We noted differences in concentrations of elements in serum and/or blood clots based on season for Ba, Be, Co, Cu, Fe, Sr, and Zn. Subsequent correlation analysis of elements with biomarkers confirmed a direct positive association of Al, Ba, Be, Fe, Sr, and Ni with the content of liver enzymes, triglycerides, total proteins, and urea in the summer season. This means that with an increased content of these elements, there is an increase in these biomarkers in the blood serum, which we evaluate negatively, especially from the point of view of possible liver damage. In the autumn season, we found a weaker negative effect of elements (Be, Fe, Zn) on liver damage parameters (AST, ALT, ALP, cholesterol, TP), which could be caused by a higher content of essential Cu in the serum in this season, as we recorded a negative correlation of Cu with the content of AST, ALT, and urea. Cu also associated a decrease in reactive oxygen species (ROS) in the summer season, which we rate as highly positive in terms of possible cellular protection against oxidative stress. In the future, we would recommend continuing a similar type of study at other locations, especially with intensive industrial and agricultural activity, or in former mining locations, as well as in breeding ponds, where it is definitely necessary to monitor the concentrations of toxic and essential elements, but also other types of environmental pollutants such as persistent organic pollutants (POPs), which represent risks not only for animals but especially for humans. Another recommendation is to test a larger number of individuals in a group, as well as multispecies studies. However, there may be a problem with the interpretation of the results due to species specificities, such as the tolerance of the species to toxicants or other physiological mechanisms. Analyses of the tissues (gills, kidneys, and liver) of freshwater fish can also provide a deeper evaluation of the impact of toxic environmental elements. We assume that it will be possible to continue experimentally in this area in the future.

## Data Availability

The authors declare that the datasets used or analysed during the current study are available from the corresponding author on reasonable request.
